# Short-Term, Voluntary Exercise Affects Morpho-Functional Maturation of Adult-Generated Neurons in Rat Hippocampus

**DOI:** 10.3390/ijms23126866

**Published:** 2022-06-20

**Authors:** Davide Lattanzi, David Savelli, Marica Pagliarini, Riccardo Cuppini, Patrizia Ambrogini

**Affiliations:** Department of Biomolecular Sciences, University of Urbino Carlo Bo, I-61029 Urbino, Italy; davide.lattanzi@uniurb.it (D.L.); david.savelli@uniurb.it (D.S.); m.pagliarini1@campus.uniurb.it (M.P.); riccardo.cuppini@uniurb.it (R.C.)

**Keywords:** adult neurogenesis, dentate gyrus, running wheel, dendritic tree complexity, newborn cell migration, synaptic plasticity, rat

## Abstract

Physical exercise is a well-proven neurogenic stimulus, promoting neuronal progenitor proliferation and affecting newborn cell survival. Besides, it has beneficial effects on brain health and cognition. Previously, we found that three days of physical activity in a very precocious period of adult-generated granule cell life is able to antedate the appearance of the first GABAergic synaptic contacts and increase T-type Ca^2+^ channel expression. Considering the role of GABA and Ca^2+^ in fostering neuronal maturation, in this study, we used short-term, voluntary exercise on a running wheel to investigate if it is able to induce long-term morphological and synaptic changes in newborn neurons. Using adult male rats, we found that: (i) three days of voluntary physical exercise can definitively influence the morpho-functional maturation process of newborn granule neurons when applied very early during their development; (ii) a significant percentage of new neurons show more mature morphological characteristics far from the end of exercise protocol; (iii) the long-term morphological effects result in enhanced synaptic plasticity. Present findings demonstrate that the morpho-functional changes induced by exercise on very immature adult-generated neurons are permanent, affecting the neuron maturation and integration in hippocampal circuitry. Our data contribute to underpinning the beneficial potential of physical activity on brain health, also performed for short times.

## 1. Introduction

Neuroplasticity is an umbrella term that includes all the functional and structural changes occurring within a neural circuit. These changes are related to functional modifications and have tremendous relevance under physiological and pathological conditions [1]. A large number of studies show that early aversive experiences in children occurring in a specific critical period definitively affect subsequent brain development [2,3]. Physical activity in children influences maturation of cognitive abilities [4,5], possibly changing neural network morpho-functional development. However, it is not clear whether acute physical activity causes long-term effects on subsequent network development.

In this context, the hippocampal region has been attracting great attention in the neuroscience research field because of its extraordinary degree of neuroplasticity. Indeed, in this brain structure, neuronal plasticity phenomena, such as synaptic long-term potentiation (LTP) and adult neurogenesis, occur, deeply affecting hippocampal functions. Notably, adult neurogenesis represents a fascinating example of plasticity that takes place in a specific hippocampal area called the dentate gyrus (DG). Here, new granule cells are daily generated and incorporated into the existing network throughout adulthood in mammals [6,7,8].

Hippocampal adult neurogenesis is a dynamic process highly dependent on the activity of the neural network. Therefore, consistently with the fact that DG receives various afferences from multiple brain regions, adult-born neuron development steps are regulated by numerous factors related to global and local neuronal activities. Voluntary physical exercise is one of the most studied activities able to positively influence adult neurogenesis. Neurogenesis improvement induced by physical activity [9,10] is considered able to maintain brain health; indeed, a large body of literature brings evidence about the beneficial consequences of physical and mental training on cognitive performance [11]. Molecular mechanisms underlying the effect of physical exercise on new neuron production are possibly related to many factors, such as BDNF [12,13], serotonin (5-hydroxytryptamine—5HT) [14,15,16], vascular endothelial growth factor (VEGF) or insulin-like growth factor-1 (IGF-1) [17,18,19,20,21].

In this scenario, we previously pointed out that a brief period of three days of physical activity delivered in a very precocious step of adult-generated granule cell development is able to antedate the appearance of the first GABAergic synaptic contacts [22,23]. Indeed, we demonstrated in rats that after voluntary physical activity on a running wheel, about 26% of 7-day-old granule cells clearly display a GABAergic contact, which is normally not seen at this early stage of new neuron development. Moreover, this very protocol was also capable of increasing the number of 7-day-old immature granule neurons showing T-type Ca^2+^ channels [22]. These findings are of particular interest because they may be correlated with the increased survival probability of newly generated granules seen in association with physical activity [24], and, considering the role of GABA and Ca^2+^ in fostering neuronal maturation and development, might have important implications in granule cell maturation [23,25,26]. From a morphological perspective, we brought evidence that the exercise induces the protrusion from 7-day-old immature neuron soma of a significantly higher number of primary dendrites without changing the total length and complexity degree of the dendritic trees [22]. In addition, a contribution of neuronal-activity-induced BDNF release in mediating the effects of the exercise has been shown [22]. This correlation is of particular interest since the neurotrophin BDNF has been implicated in activity-dependent synaptic plasticity and network remodeling [27,28]. BDNF is able to regulate the extent of adult hippocampal neurogenesis [29], presumably via its specific TrkB receptors [30], which are expressed on proliferating neural progenitor cells in the dentate gyrus [31], suggesting a direct influence of BDNF on neurogenesis. Consistently, we found that the TrkB agonist 7,8-dihydroxyflavone mimics the effect of physical exercise in rats kept under control condition, while the TrkB antagonist ANA-12 counteracts the effect of the three-days voluntary running [22].

Considering the results described above, we decided to go further and evaluate the long-term effects on neuron plasticity of the same physical activity protocol consisting of three days of voluntary wheel running administered at a very early stage during new neuron development. Thus, in the present work, we moved our attention to 30-day-old adult-generated granule cells, where we performed morphological and electrophysiological analyses in order to assess if a short-term voluntary exercise is able to induce long-term plastic morphological and synaptic changes, persistently affecting hippocampal function.

## 2. Results

Rats included in the RUN group, housed in cages containing stainless steel running wheels equipped with an electronic counter, ran voluntarily and primarily during the dark period (night). An average distance of 2.46 ± 0.68 km/day was covered by rats. In general, the time spent running by rats increased night by night over the three days of exercise, reaching an overall average distance of 7.4 ± 0.68 km. The inter-individual variation in running activity was relatively small, and no rats were inactive.

### 2.1. Electrophysiological Analysis 

**Figure 1:**
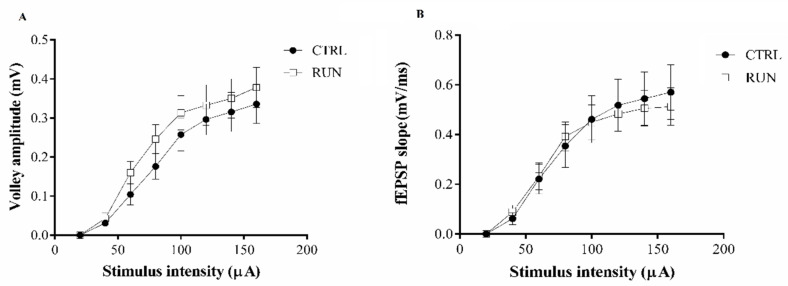
Basal synaptic transmission evaluation. Input-output of stimulus plots versus fiber volley amplitude (**A**) and stimulus versus fEPSP slope (**B**) in CTRL and RUN group. Field potential and fiber volley amplitude responses to increasing intensity stimuli of the perforant pathway were not significantly different in the two groups.

Field recordings, in transversal hippocampal slices were used to investigate synaptic plasticity in the dentate gyrus of CTRL and RUN rats by evaluating basal synaptic transmission (input/output curves) and the ability to elicit LTP. The field potential responses to increasing intensity stimuli of the perforant pathway were not significantly different in the two groups (Figure 1A). Similarly, the relationship between the fiber volley amplitude and the stimulus intensity did not show differences between groups (Figure 1B). 

**Figure 2:**
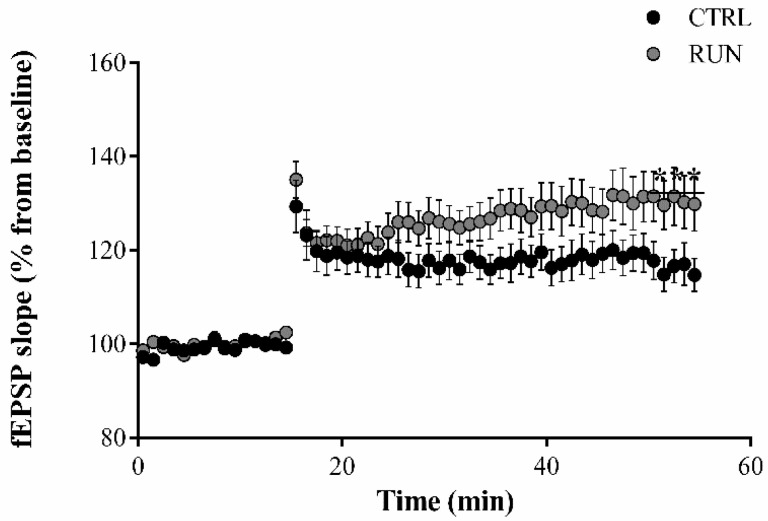
Electrophysiology analysis of long-term potentiation (LTP). The slope of EPSPs resulting from the parasagittal sections (400 µm) of the hippocampal region was analyzed. The slopes of the EPSP in the RUN group were significantly increased compared to CTRL group after recording for 40 min following high-frequency stimulation; in addition, the y-intercept in the last 10 min of recording was significantly higher in RUN group. Data statistical analysis: linear regression slope F = 7.09361, *p* < 0.01; y-intercept F = 32.0045, *p* < 0.0001.

High-frequency stimulation of the perforant pathway elicited a robust LTP both in CTRL and in RUN groups (Figure 2). In slices from the RUN group, the evoked LTP was similar to that elicited in the CTRL group during the first 15 min, but fEPSP in RUN animals reached significantly higher values than controls, especially in the last minutes of recordings (Figure 2). 

**Figure 3:**
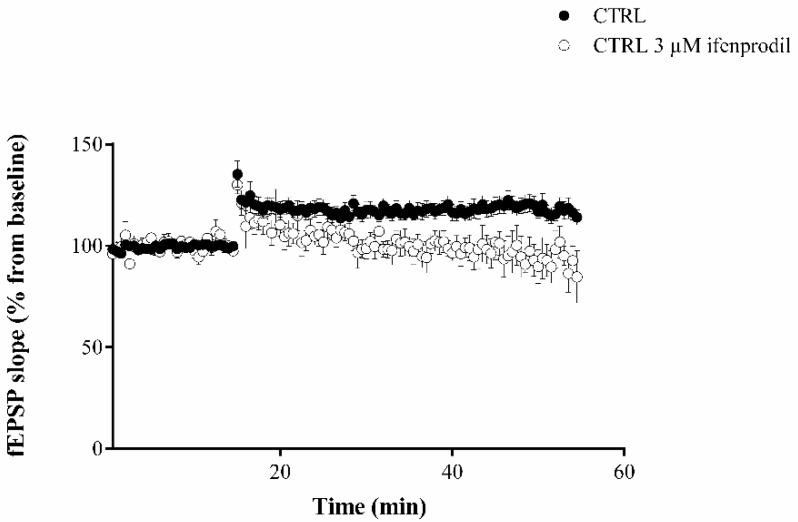
Long-term potentiation (LTP) in presence of ifenprodil. The slopes of the EPSP evoked by high-frequency stimulation in CTRL slices treated with 3 µM ifenprodil significantly decreased after recording for 40 min following; in addition, the y-intercept in the last 10 min of recording was significantly lower in the group treated with 3 µM ifenprodil group. Data statistical analysis: linear regression slope F = 128.141, *p* < 0.0001; y-intercept F = 566.889, *p* < 0.0001.

In accordance with the literature [32], ifenprodil, which inhibits NR2B-containing NMDA receptors—almost exclusively expressed in immature cells—prevented the synaptic potentiation after the tetanic stimulation of the perforant pathway (Figure 3). This finding indicates that immature granule cells, affected by three days of physical exercise in a precocious period of their development, contribute to the improved LTP maintenance observed after the high-frequency stimulation of the perforant pathway, which induces a synaptic potentiation based on immature cells. 

### 2.2. Morphological Analysis

**Figure 4:**
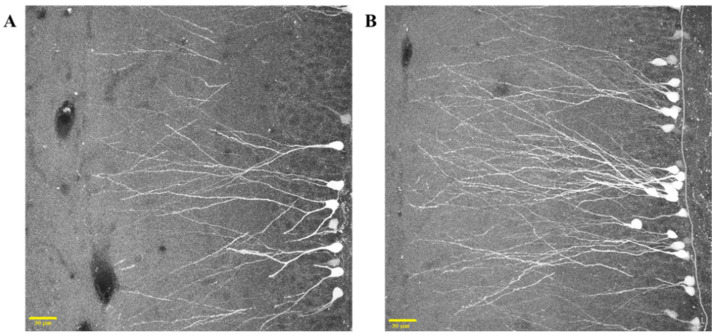
Retroviral labeling of 30-day-old dentate granule cells in CTRL (**A**) and RUN (**B**) rats. Morphological reconstruction of each GFP-positive cell shows retrovirally labeled newborn dentate granule cells expressing GFP throughout the entire cell. Scale bar, 30 μm.

In vivo injection of GFP-expressing retrovirus allowed us to later reveal, in transversal hippocampal slices, several newly-generated GFP-positive cells located in the dentate gyrus of CTRL and RUN rats. In general, labeled cells of both groups showed one or more primary dendrites, which emerged from the top of the cellular soma and branched into higher-order dendrites (Figure 4A,B). The dendrites reached the molecular layer of DG, and the spines were noticeable. 

**Figure 5:**
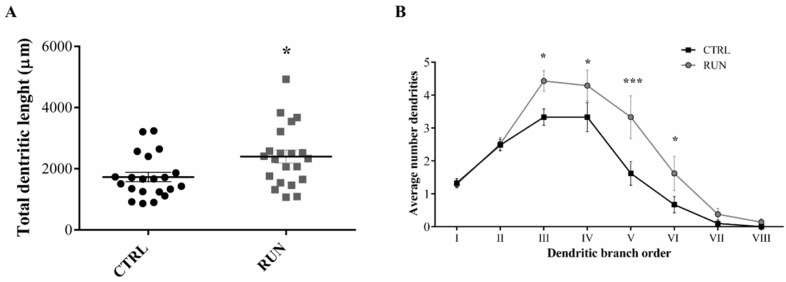
Morphological properties of 30-day-old granule cells. Comparison of total dendritic length (**A**) and dendritic tree complexity (**B**) between GFP-positive neurons of rats belonging to CTRL group (n = 21 cells) and RUN group (n = 21 cells). Statistical analyses: (**A**) unpaired Student’s *t*-test * *p* < 0.05; (**B**) multiple Student’s *t*-test * *p* < 0.05, *** *p* < 0.001. All data are expressed as mean ± SEM.

The measurement of the total dendritic length pointed out that dendrite extension was significantly higher in the RUN group than in CTRLs (Figure 5A). Moreover, despite the similar number of the I order dendrites between the two groups, a significantly higher number of III to VI order dendrites was observed in the RUN group (Figure 5B), suggesting higher dendritic complexity trees in the RUN group, as confirmed by Sholl’s analysis. 

**Figure 6:**
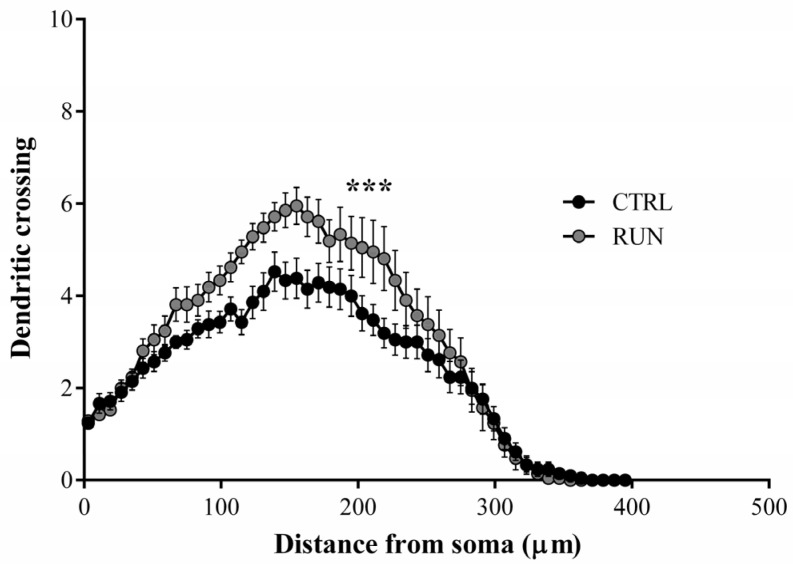
Sholl’s analysis of dentate gyrus GFP-positive neurons. Dendritic length measured by Sholl’s analysis (superimposed ring 8 μm) revealed an increase in arborization, particularly evident at distances between 115–227 μm from the soma in the RUN group. Data statistical analysis: multiple Student’s *t*-test, *** *p* < 0.001 from 115 to 227 µm. All data are expressed as mean ± SEM, CTRL n = 21, RUN n = 21.

In particular, the most remarkable differences between groups were visible in the area extending from 115 to 227 micrometers away from the cell body (Figure 6). 

**Figure 7:**
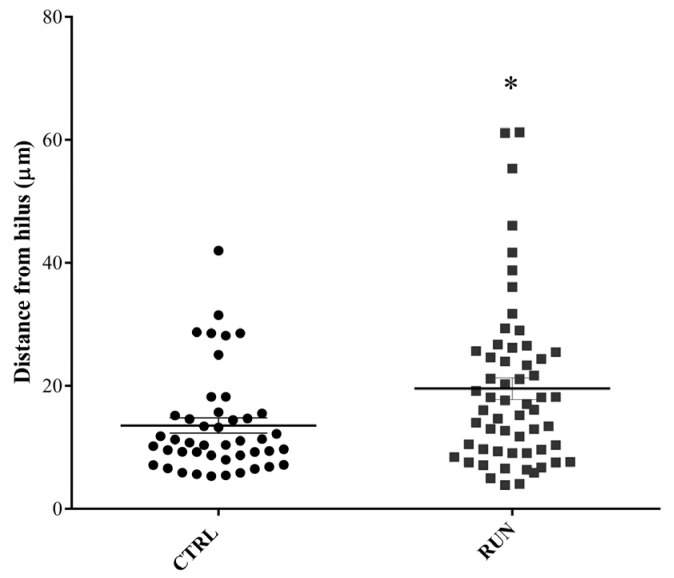
Adult-generated granule neurons migration assessment. Newborn granule cell body position within the granule cell layer was analyzed by measuring the distance between each GFP-positive cell body and the SGZ/hilus zone. Many newborn granule cells in the dentate gyrus of the RUN group migrated farther from the considered zone compared to controls. Data statistical analysis: unpaired Student’s *t*-test, * *p* < 0.05. All data are expressed as mean ± SEM, CTRL n = 44, RUN n = 57.

Along the DG granule differentiation process the adult-generated neurons migrate throughout the granule cell layer; thus, the distance of the GFP-positive cell body from the SGZ/hilus was calculated to gain further insight into the newborn granule maturation level under control and exercised conditions. From this evaluation, we found that new granule neurons of the RUN group were generally farther from the SGZ/hilus in comparison to the CTRL group (Figure 7). 

**Figure 8:**
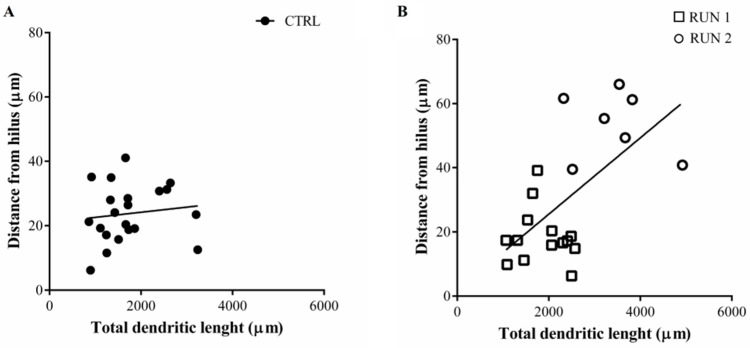
Scatterplot of cluster assignments in CTRL (**A**) and RUN (**B**) groups projected on distance from SGZ/hilus and total dendritic length parameters. The clusters are represented by different shapes or colors (CTRL●, RUN 1 □, RUN 2 ◦). Cluster ranking score was calculated automatically by Tableau software. Statistical analysis: distance from hilus (µm) RUN 1 vs. RUN 2, F = 14.08, *p* < 0.01; total dendritic length (µm) RUN 1 vs. RUN 2, F = 10.94, *p* < 0.01. Note that in the RUN group, there was a significant correlation between total dendritic length and distance from hilus (*p* = 0.0029).

It is noteworthy that in plotting the cell body distance from the SGZ/hilus versus the dendritic length, it was possible to distinguish two separate populations of labeled cells in the RUN group that we named RUN1 and RUN2 (Figure 8B). The presence of subpopulations was not appreciable in the CTRL group (Figure 8A).

**Figure 9:**
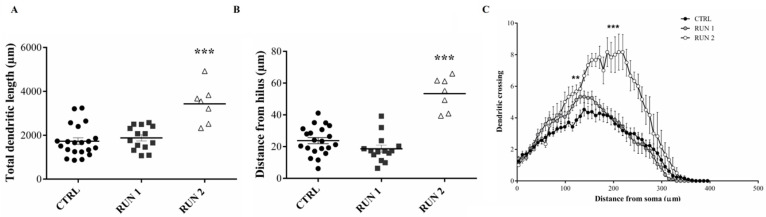
Morphological properties of CTRL, RUN 1, and RUN 2 30-day-old granule cells. Comparison of total dendritic length (**A**), distance of neuronal body from hilar border (**B**), and dendritic tree complexity (**C**) between CTRL, RUN1, and RUN2 GFP-positive neurons. Statistical analyses: (**A**) unpaired Student’s *t*-test *** *p* < 0.001; (**B**) unpaired Student’s *t*-test *** *p* < 0.001; (**C**) two-way ANOVA Tukey’s multiple comparisons test. CTRL vs. RUN1 *** *p* < 0.001 from 115 to 131 µm, CTRL vs. RUN2 *** *p* < 0.0001 from 99 to 267 µm, RUN1 vs. RUN2 ** *p* < 0.01 from 155 to 267 µm. All data are expressed as mean ± SEM. CTRL n = 21, RUN1 n = 14, RUN2 n = 7.

As expected, the GFP-positive cells located farther from the SGZ/hilus (RUN2) showed the greatest dendritic length (Figure 9A) and the most complex dendritic tree (Figure 9B,C). Therefore, it emerged that the differences between CTRL and RUN groups were mainly due to a subpopulation of cells detected in the latter group only. Notably, part of the GFP-positive granule cells in the RUN group (RUN1) were not different in total dendritic length and distance from SGZ/hilus when compared to labeled cells in CTRLs (Figure 9A); on the other hand, about 33% of the RUN newborn cells (RUN2) were characterized by a greater total dendritic length and distance of migration (Figure 9A,B). In addition, data from Sholl’s analysis, which provides an estimate of dendrite arborization by evaluating the dendritic crossing along the Sholl rings, revealed a significantly higher degree of arborization in dendrites of the RUN2 subpopulation compared to the CTRL and RUN1 groups (Figure 9C). RUN1 new neurons also exhibited an increased dendritic arborization with respect to CTRL, but it was limited to their proximal segments, where no difference was found compared to the RUN2 neuronal subpopulation.

## 3. Discussion

**Figure 10:**
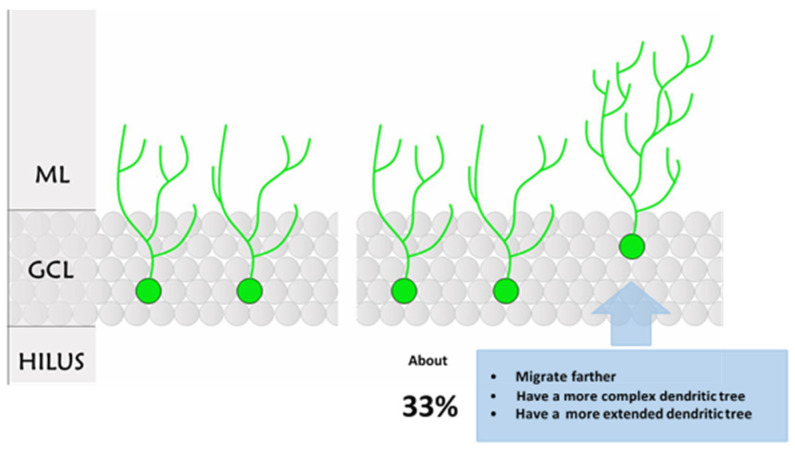
Summary representation of the differences between CTRL and RUN 30-day-old GFP-positive granule cells. In the figure are outlined the features of newly generated granule cells at 30 days after GFP injection in CTRL vs. RUN groups. ML: molecular layer; GCL: granule cell layer.

The present study aimed to gain insight into the long-term effects of a short-term, voluntary running exercise on the development of adult-generated neurons in the DG of rat hippocampus. The key findings in our study are as follows: (i) three days of voluntary physical exercise definitively influence the morpho-functional maturation process of newborn granule neurons when applied very early during their development; (ii) indeed, a subpopulation of new neurons shows more mature morphological characteristics (Figure 10 for a summary of features), highlighting that long-term effects are targeted in selected cells; (iii) the long-term morphological effects are paralleled by enhanced synaptic plasticity, possibly affecting hippocampal functions.

Physical exercise was ascertained to affect hippocampal neurogenesis and synaptogenesis [33,34,35], but its effect may depend on several parameters, such as type, duration, and intensity of exercise [36]. About this, it has been demonstrated that long-term running, mainly voluntary running, can significantly enhance cell proliferation, neuronal survival, and synaptic activity in the rat hippocampus. Importantly, elevated neurogenesis induced by voluntary exercise has been associated with some form of cognitive improvement [37,38,39]. 

In this scenario, we found that even a short-term voluntary running, only lasting three days, is sufficient to positively influence the morpho-functional development of newly-generated granule cells, with possible implications on hippocampal functions. Notably, new cells in DG were responsive to the hippocampal activation induced by voluntary exercise very early after their birth, and part of them showed morphological modifications when analyzed far from the end of exercise. Indeed, an increased dendritic tree complexity and length, together with enhanced migration, have been found in about 33% of 30-day-old GFP-positive cells of the RUN group, indicating more mature neuronal characteristics. Interestingly, this percentage is very close to our previous findings within the 7-day-old cell population, which received a precocious GABAergic contact after the same voluntary running protocol applied here [22]. It is, therefore, feasible that the anticipation of the synaptic GABAergic contact, which antedates the exposition of cells to GABA action, might affect the survival and morpho-functional development of granules that receive the contact, and speed up neuronal differentiation. In keeping with this hypothesis, we previously highlighted a mechanism through which GABA fine-tunes intracellular calcium homeostasis in rat adult-born granule neurons at a very early stage of their maturation [26]. Thus, considering the crucial role played by calcium signaling in neurodevelopment [40], it is conceivable that GABA activity related to physical exercise hippocampal activation may influence neuron developmental processes, such as dendritic arborization, neuronal migration [41,42,43], as well as cell survival, by modulating intracellular Ca^2+^ transients.

The advanced neuronal morphological differentiation of the 30-day-old cell subpopulation, in particular the increased extension and branching of the dendritic trees, underpins the possibility of a larger number of synaptic contacts onto them, resulting in a deeper integration into the hippocampal network with a possible implication in the hippocampal functions. In particular, this more mature population, which, however, is still in the “critical period” of high excitability [44,45,46,47,48] due to a depolarizing GABAergic input, might influence the synaptic plasticity related to the pool of immature cells [32]. According to this hypothesis, our data derived from the electrophysiological analysis of field potentials revealed a difference in the maintenance phase of LTP induced through high-frequency stimulation of the medial perforant pathway. In the RUN group, this phase of the synaptic potentiation is characterized by higher fEPSPs, suggesting a link between voluntary running, enhanced morphological development, and hippocampal functions. 

The hippocampus is a crucial structure for episodic memory, for the establishment of memory traces about “where”, “how”, and “when” a precise event happened, and the DG of the hippocampus is known to have a role in pattern separation, by involving newborn neurons [49]. Pattern separation is a key neuronal process relevant to cognitive functioning, which can be influenced by exercise in several different ways [50]. This function of DG underlies cognitive aspects of memory coding, allowing discrimination of similar experiences or objects [51] (dorsal DG), while its impairment may underlie affective disorders, including depression, anxiety, and post-traumatic stress disorder [52] (ventral DG). In the DG, adult-born granule cells pass through a “critical period” of their development characterized by morpho-functional properties, specifically hyperexcitability due to the reverse driving force of Cl^−^ and, consequently, the excitatory effect of GABA [32], suited for pattern integration function and temporal separation of memories [53,54]. As suggested by Piatti et al. [55], since the higher number of immature granules in the development period is suited to perform pattern integration function, the increased development speed of immature granules might ameliorate temporal resolution and the reliability of multiple-event memorization. In addition, variation in the dendritic trees’ complexity and extension can allow a higher number of synaptic contacts in the immature population; all of these contacts originate from not only the entorhinal cortex, but also from CA3 back-projection [56] and might play an important role in the pattern integration function, supporting the correlation between similar events or comparable stimuli. It is, therefore, suggested that the subpopulation of more mature newly-generated neurons found in the RUN group can allow a deeper integration into the hippocampal network. The effects related to physical activity can influence aspects related to memory, such as the capability to associate different mnestic traces or discern similar memory traces established in different points close in time. Thus, the difference found in the maintenance phase of LTP is probably related to the morphological characteristics of this more mature subpopulation of 30-day-old cells and might influence DG functions leading to physical activity-associated improvements in cognitive performance widely reported in the literature [57,58,59,60]. This difference might lead to improvement in the pattern integration function of the DG in the RUN group since it could improve the association of events through the activation of a similar population of immature granules, which, after the completion of their development and reduction in excitability, will only respond to the events experimented on during their development [54].

As regards the mechanistic aspect, BDNF seems to be the principal factor in mediating the effects of physical activity on neurogenesis, and its mRNA and protein levels increase in the hippocampus after exercise [61]. Consistently, our previous data [22] show that the morpho-functional effects induced by the same short-term voluntary running protocol on 7-day-old adult-generated cells in DG are mediated by BDNF, acting through TrkB receptors via the MAP/ERK pathway and CREB activation [62,63]. In turn, BDNF expression can be increased via a MAPK-CREB-dependent mechanism that, in developing neurons, can be induced by GABA excitatory action [64]. It is documented that the CREB transcription factor plays a major role in synaptic plasticity [65,66], synaptogenesis, and newborn neuron integration in the adult hippocampus by inducing miRNA expression, such as miR-132 [67,68,69]. Accordingly, Mojtahedi and colleagues [70] found that voluntary running has the most positive impact on biomarkers, such as miRNA, that are associated with stimulating neurogenesis and synapse formation in the rat hippocampus.

Summing up, based on our results and taking into account the above consideration, we can figure out the following scenario: short-term voluntary running increases hippocampal activity and influences adult-generated newborn granule cells very early during their development, promoting the precocious appearance of the first GABAergic synaptic contacts onto them, possibly by inducing BDNF release and TrKB receptor activation and regulating miRNA expression involved in neurogenesis processes. The anticipation of synaptogenesis fosters newborn cell survival and differentiation, speeding up their integration into the hippocampal network. The population of adult-generated young neurons affected by voluntary exercise enhances hippocampal plasticity, thus possibly improving hippocampal functions.

In conclusion, other authors have reported beneficial effects of short-term exercise on neurogenesis processes [71,72], but in our study, we demonstrate not only that the morpho-functional changes induced by exercise on very immature adult-generated neurons are permanent, being detected far from the end of exercise, but also that exercise changes timing and kind of morpho-functional development occurring in the period of time subsequent the end of exercise. Therefore, the present findings contribute to increasing the body of information on exercise-induced changes and underpinning the beneficial potential of physical activity on brain health, also when it is performed for short times.

## 4. Materials and Methods

### 4.1. Animals and Running Protocol

Sprague Dawley (SD) rats (Charles River Laboratories, Italy) were reared in a temperature (21 ± 1 °C) and humidity (50 ± 5%)-controlled vivarium room with a 12 h/12 h light/dark cycle (6:00 a.m. to 6:00 p.m. lights on). The animals were group-housed in standard cages with water and food ad libitum. Male rats (6–8-week-old, n = 27, from different litters) were used in accordance with the Italian law on animal experimentation (D.lgs. 26/2014; research project permitted with authorization N. 465/2015-PR by the Italian Ministry of Health). All efforts were made to minimize animal suffering and to reduce the number of animals used.

To in vivo label adult newly-generated granule cells in hippocampal DG, rats were anesthetized with sodium thiopental (45 mg/Kg body weight) and stereotaxically injected with Green Fluorescent Protein (GFP)-expressing retrovirus, as previously described [23]. Briefly, retroviral GFP-expressing virions were obtained cotransfecting the ecotropic packaging Phoenix cell line (ORBIGEN) and infused bilaterally (twice 30 min spaced) into the dentate gyrus (1 μL at 0.5 μL per min) (anteroposterior: 23 mm from bregma; lateral: 2 mm; ventral: 3.2 mm). 

On the fourth day after surgery, the animals were randomly assigned to the following experimental groups: 1. voluntary running rats housed in a wheel cage (RUN; running for three days: 4°, 5°, and 6° day after retroviral injection); 2. control rats (CTRL) housed in a standard cage and not exposed to any behavioral experience. 

### 4.2. Electrophysiological Experiments 

#### 4.2.1. Slices Preparation 

Thirty days after the retroviral injection, rats were anesthetized with ketamine (65 mg/Kg b.w.) and killed by decapitation. Brains were quickly removed and incubated in chilled oxygenated solution containing in millimolar: 110.0 choline Cl^−^, 2.5 KCl, 1.3 NaH_2_PO_4_, 5.0 NaHCO_3_, 0.5 CaCl_2_, 7.0 MgCl_2_, 20.0 dextrose, 1.3 Na^+^ ascorbate, 0.6 Na^+^ pyruvate, 5.5 kinurenic acid (pH: 7.4; 320 mOsm). Hippocampal transversal slices (400 μm thick) were obtained from each hemisphere by vibrating microtome (Campden Instruments) and allowed to recover in oxygenated Artificial Cerebrospinal Fluid (ACSF) containing in millimolar: 125.0 NaCl, 2.5 KCl, 1.3 NaH_2_PO_4_, 25.0 NaHCO_3_, 2.0 CaCl_2_, 1.3 MgCl_2_, 1.3 Na^+^ ascorbate, 0.6 Na^+^ pyruvate, 10.0 dextrose (pH: 7.4; 320 mOsm). The slices were kept in ACSF solution for at least 1 h at room temperature before electrophysiological recordings. Afterwards, slices were individually transferred into a recording chamber where they were held in place with nylon mesh and continuously superfused throughout the electrophysiological recordings with oxygenated ACSF at a rate of 3 mL/min. 

#### 4.2.2. Electrophysiological Field Recordings

The influence of three days of voluntary running on synaptic plasticity was investigated, evaluating the ability of DG granules to elicit LTP after the high-frequency stimulation (HFS) of the medial perforant pathway in CTRL (n = 9 rats) and RUN (n = 10 rats) groups. To specifically assess the role of immature granule cells, which were affected by the training protocol applied [23], a peculiar stimulation protocol, developed by Snyder and colleagues [32] and able to elicit LTP on the sole immature granule cells, was applied. 

To this purpose, recording and bipolar stimulating electrodes were prepared and filled with ACSF. The recording electrode was placed in the molecular layer of the DG, while the stimulating one was in the medial perforant pathway. The stimulation intensity that produced a half-maximal response was chosen for test pulse and tetanic stimulation. Slices giving extracellular field excitatory postsynaptic potentials (fEPSPs) of at least 1 mV in amplitude were considered for recordings. Low-frequency test pulses (at 30 s intervals) were applied to elicit baseline responses. Once obtained, a stable baseline of approximately 20 min, the medial perforant pathway was simulated applying the LTP protocol consisting of 2 trains, 500 ms each, 100 Hz within the train, repeated every 20s. The fEPSP was then monitored by recordings for 40 min. Slope (between 10% and 80% of max) of the fEPSP was analyzed and taken as a measure of synaptic strength. Values were normalized to the mean value obtained over the last 20 min of the baseline period and expressed as a percent of this baseline value. 

To confirm that the elicited LTP was due to the immature granule cells only, as NMDARs containing NR2B subunits are preferentially expressed on immature granule cell membranes [73], some slices underwent the LTP protocol in the presence of 3 μM ifenprodil, an NMDAR antagonist that selectively inhibits receptors containing the NR2B subunit, in the perfusion ACSF.

### 4.3. Morphological Analysis

To investigate if the voluntary running induced long-term structural modifications in newly-generated granule cells born during the training, a morphological study of 30-day-old GFP-positive DG cells was performed on slices from the RUN (n = 4 rats) and CTRLs (n = 4 rats) groups. The slices, obtained as described above, were immediately fixed in paraformaldehyde 4% in 0.1 M phosphate buffer saline (phosphate buffered saline (PBS), pH = 7.4) and kept overnight at 4 °C. To enhance GFP labeling, free-floating slices were immunohistochemically processed by incubating overnight at 4 °C with the anti-GFP made in mouse primary monoclonal antibody (1:200 in PBS; Sigma) followed by the FITC-conjugated horse anti-mouse IgG secondary antibody (1:50 in PBS; Vector, D.B.A.). 

**Figure 11:**
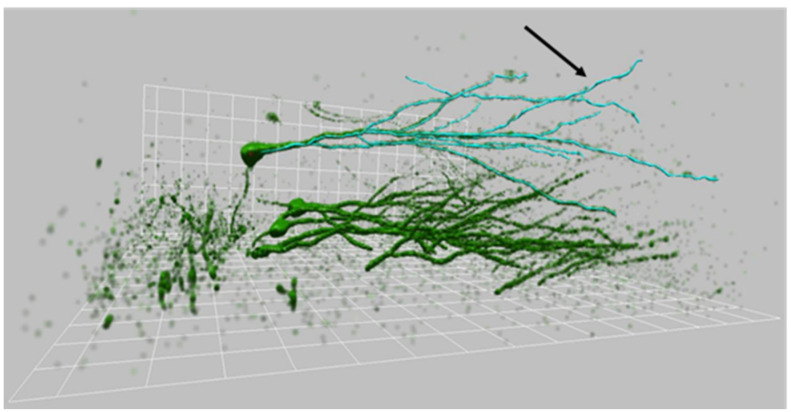
Three-dimensional reconstruction of granule cells. Three-dimensional reconstruction of 30-day-old GFP-positive granule cells performed using NeuronStudio software on confocal microscopy stacks. It is possible to notice the blue traces of dendrites (arrow) which spread through the three dimensions of the slice.

GFP-positive cell was observed and acquired using a Leica TCS-SL confocal microscope equipped with Argon and He/Ne laser sources. The reconstruction of each GFP-labeled granule cell has been performed using NeuronStudio software, following the dendritic arborization through the three dimensions of the slice thickness made of confocal stacks. Morphological analysis was carried out on a subset of reconstructed cells showing no clear dendritic cutting at the slice surface (Figure 11). 

The images obtained were used to evaluate the total length of dendrites and the number of primary dendrites. To evaluate dendritic arborization, the images obtained in NeuronStudio were analyzed using NeuronJ. Sholl’s analysis was adopted to estimate dendrite arborization and was performed by Sholl’s Analysis Plugin (https://imagej.net/plugins/sholl-analysis, accessed on 17 June 2022) [74], using an 8-μm interval between concentric circles. Moreover, considering that throughout the development stages the newly-generated DG granules move from the subgranular zone (SGZ), located at the border of the granule cell layer facing hilus, where they originate, through the granular layer, the distance of each GFP-positive granule cell from the SGZ/hilus has been evaluated as an index of neuronal migration. 

### 4.4. Statistical Analysis 

Data are expressed as the mean ± SEM. Statistical analyses were performed using the commercial program GraphPad PRISM version 6.0.1 (GraphPad Software, San Diego, California, USA) by appropriately applying linear regression analysis, multiple Student’s *t*-test, one-way ANOVA with Tukey post-hoc test, and two-way ANOVA with Tukey’s post-hoc test. Cluster analysis was performed using Tableau analysis software version 2021.2 (TABLEAU analysis software, Seattle, Washington, USA). Tableau uses the K-means algorithm for clustering. Starting with a cluster, the method chooses a variable whose average value is used as a threshold to divide the data in two. The centroids of these two parts are then used to initialize K-means to optimize the membership of the two clusters. The significance threshold was established at *p* = 0.05.

## Data Availability

Not applicable.
